# Early hyperoxia and 28-day mortality in patients on venoarterial ECMO support for refractory cardiogenic shock: discussion about potential confounding factors

**DOI:** 10.1186/s13054-022-04181-z

**Published:** 2022-10-17

**Authors:** Hadrien Winiszewski, Gael Piton, Gilles Capellier

**Affiliations:** 1 Service de Réanimation Médicale, CHU, Besançon, France; 2 Department of Epidemiology and Preventive Medicine, Faculty of Medicine, School of Public Health and Preventive Medicine, Nursing and Health Sciences, Clayton, Australia; 3 Research Unit EA 3920 and SFR FED 4234, University of Franche Comté, Besancon, France

**To the Editor,**

We would like to commend Moussa et al. for their work recently published in Critical Care [1] in which they demonstrated an association and a dose–response between early hyperoxemia and 28-day mortality in patients with refractory cardiogenic shock supported by veno-arterial extracorporeal membrane oxygenation (VA-ECMO). While several studies have previously reported such association in the setting of extracorporeal cardiopulmonary resuscitation, previous studies enrolling patients with refractory cardiogenic shock have reported conflicting results [2].

Although we think, as the authors, that the harmful effect of hyperoxemia in these very sick patients exposed to ischemia and reperfusion is probable, we would like to discuss some possible confounding factors.

First, the association between right arm hyperoxemia and prognosis reported in observational studies might be impacted by the native cardiac function. Indeed, during femoro-femoral VA-ECMO, the right arm P_a_O_2_ is determined by both S_v_O_2_, hemoglobin concentration, native lung function, inspired oxygen fraction on ventilator (F_I_O_2_), positive end expiratory pressure, sweep gas oxygen fraction (F_S_O_2_), and the balance between native cardiac function and ECMO blood flow. The later determines the mixing zone location, *i.e.*, the zone in the aorta where the blood ejected from the heart and oxygenated by the lungs meets the blood ejected and oxygenated by the ECMO [2]. Because of the oxygenator’s performance, postoxygenator oxygen partial pressure (P_POST_O_2_) can rise to 500 mmHg. Then, hyperoxemic patients might be those for whom right arm P_a_O_2_ is mainly determined by the ECMO when the mixing zone is in the aortic arch close to the brachiocephalic trunk. Such situation might occur in case of high ECMO blood flow and severe cardiac failure, which could lead per se to a poor prognosis.

To explore this possibility, the authors have tested the interaction between ECMO blood flow and right arm P_a_O_2_ on admission. Although they did not find any association, we cannot rule out that mean daily peak P_a_O_2_, absolute peak P_a_O_2_, and overall mean P_a_O_2_ could be associated with ECMO blood flow. It could be also of interest to test the association between right arm P_a_O_2_ and surrogates of native cardiac function, such as pulse pressure or end tidal CO_2_ [3].

Another way to support the causality link between hyperoxemia and poor prognosis is to demonstrate that exposition to oxygen is different between survivors and non-survivors. In Table 2, it is unclear if the “F_I_O_2_” corresponds to the inspired oxygen fraction on the ventilator, or to the F_S_O_2_ set on the ECMO. It could be of interest to precise both F_I_O_2_ and F_S_O_2_. If “F_I_O_2_” actually corresponds to F_S_O_2_, we can hypothesize that P_POST_O_2_ was higher in non-survivors than in survivors, which potentially has resulted in more reperfusion injury of intra-abdominal organs mediated by radical oxygen species [4].

Finally, the number of arterial blood gas (ABG) analysis performed during the 48 first hours of VA-ECMO support might be an interesting data to show to ensure that different overall mean P_a_O_2_ reflect different oxygen exposure. Indeed, overall mean P_a_O_2_ is mathematically linked to the number of ABG sampled. If several ABG analyses are performed during a short period of hyperoxemia, overall mean P_a_O_2_ could overestimate oxygen exposure. Assuming that the most severe patients have more frequent ABG analysis, we cannot exclude that overall mean P_a_O_2_ could overestimate oxygen exposure in most severe patients. Then, it could be of interest to determine the relation between 28-day mortality and time-weighted P_a_O_2_ [5], a metric of arterial oxygenation which better reflects oxygen exposure than overall mean P_a_O_2_.

Beyond these points of discussion, Moussa et al. have to be congratulated for their work which adds rationale for the two ongoing randomized trials on oxygen management during VA-ECMO support (BLENDER NCT03841084, and ECMOXY NCT04990349).

## Data Availability

Not applicable.
