# Supplementary material for: Early hyperoxia and 28-day mortality in patients on venoarterial ECMO support for refractory cardiogenic shock: discussion about potential confounding factors
Source: Crit Care. 2022 Oct 17;26:313. doi: 10.1186/s13054-022-04181-z (PMC9578254; doi:10.1186/s13054-022-04181-z)
Supplement: Related texts

# Reply: Response

**Authors:** Mouhamed Djahoum Moussa, Christophe Beyls, Osama Abou-Arab

**Affiliations:** 4 grid.410463.4 0000 0004 0471 8845 Pôle d’Anesthésie-Réanimation, Lille Hospital University, 59000 Lille, France; 5 grid.134996.0 0000 0004 0593 702X Anesthesia and Critical Care Medicine Department, Amiens University Medical Center, 80054 Amiens, France

**Dear Dr Winiszewski,**

We would like to thank Dr Winiszewski and colleagues for their comments that raises one of the major question regarding the management of peripheral veno-arterial ECMO (VA-ECMO)—“What are the determinants of arterial oxygenation in patients receiving VA-ECMO support?”. Numerous studies have attempted to answer this question, but data are limited for VA-ECMO patients and are even more limited when only patients in cardiogenic shock are considered (excluding eCPR). Retrograde arterial blood flow and mixed blood flow zone phenomena exist only in the VA-ECMO settings, which do not allow extrapolation of data from VV to VA-ECMO.

Andrei and colleagues [6] recently provided the interesting observation that the main determinants of PaO_2_ measured at the right arm level are the blood flow delivered by the VA-ECMO and the sweep gas oxygen fraction. None of the ventilator parameters (FiO_2_, respiratory rate) were associated with right arm PaO_2_. Furthermore, this study demonstrated that during the initial phase of VA-ECMO support, the impact of the native heart function could be neglected and was not related to PaO_2_. Indeed, the participation of the native cardiac function on the PaO_2_ is modulated by the level of VA-ECMO assistance. The greater the level of VA-ECMO support, the lower the contribution of the native cardiac function. However, we usually observe during the initial phase of refractory cardiogenic shock a collapsed native heart function and a high level of VA-ECMO support (i.e., high blood flow), which suppresses the influence of the native heart on PaO_2_. This may also explain why pulse pressure, which is largely determined by the native heart function, is not associated with right arm PaO_2_. Thus, at the start of VA-ECMO support, an elevated right arm PaO_2_ may often reflect hyperoxemia throughout the body. Because our study focused on the early period of VA-ECMO support, we believe that we have avoided these confounding factors.

We agree that the time-weighted PaO_2_ may better reflect the time of exposure to hyperoxemia than two time points over a 24-h period, but this remain difficult to use in clinical practice. All patients underwent arterial blood gases according to the local centers' “standard of care”, so that we can assume that a quite similar number of PaO_2_ measurements were obtained in each patient.

This method is far from perfect, but it is clinically relevant and close to what is reported in previous studies evaluating hyperoxemia [7].

In summary, despite these limitations that were already discussed [8] and based on the above arguments, we believe that hyperoxemia is associated with a higher risk of 28-days mortality. Hopefully, further randomized clinical trials (BLENDER NCT03841084 and ECMOXY NCT04990349) will be able to answer this question.
